# Predicting Metabolic Dysfunction–Associated Fatty Liver Disease Phenotypes Among Adults: 2-Stage Contrastive Learning Method

**DOI:** 10.2196/75747

**Published:** 2025-12-12

**Authors:** Sizhe Jasmine Chen, Da Xu, Derek K Hu, Paul Jen-Hwa Hu, Ting-Shuo Huang

**Affiliations:** 1 Department of Operations and Information Systems, David Eccles School of Business, University of Utah, 1655 East Campus Center Drive, Salt Lake City, UT, United States, 1 801-587-7785; 2 Department of Marketing, Analytics, and Professional Sales, School of Business Administration, University of Mississippi, University, MS, United States; 3 Department of Biomedical Engineering and Department of Computer Engineering and Computer Science, California State University, Long Beach, Long Beach, CA, United States; 4 Division of General Surgery, Department of Surgery, Jen-Ai Hospital, Taichung, Taiwan; 5 Department of Surgery, Chang Gung Memorial Hospital, Keelung Branch, Keelung, Taiwan; 6 Department of Chinese Medicine, College of Medicine, Chang Gung University, Taoyuan, Taiwan

**Keywords:** metabolic dysfunction–associated fatty liver disease, phenotype, graph representation learning, multiview contrastive learning, predictive analytics

## Abstract

**Background:**

Metabolic dysfunction–associated fatty liver disease (MAFLD) is a leading cause of chronic disease and can progress to liver fibrosis or hepatocellular carcinoma. Its subtypes—obese, diabetic, and lean—are associated with varying degrees of fibrotic burden and different complications, yet the existing analytics methods often overlook its multisystem nature, intraphenotype variability, and disease dynamics. These limitations hinder accurate risk stratification and restrict personalized intervention planning.

**Objective:**

This study developed a novel, 2-stage, contrastive learning–based method to predict the phenotype of MAFLD among adults. This method leverages multiview contrastive learning; it models individual heterogeneities and important relationships in clinical and survey-based data to predict phenotypes among adults, thus supporting clinical decision-making and personalized care.

**Methods:**

Demographic, clinical, lifestyle, and genetic family history data of 4408 adults revealed how capturing essential relationships in patient data from different sources can transform individual-level representations into multiple, complementary views. Evaluation of the predictive efficacy of the proposed method in comparison with 8 prevalent methods relied on recall, precision, *F*_1_-score, and area under the curve values. Moreover, a Shapley additive explanation analysis was performed for interpretability.

**Results:**

The proposed method consistently and significantly outperformed all benchmark methods. It attained the highest *F*_1_-score, showing a 32.8% improvement for nondiabetic MAFLD (0.531 vs 0.400) and 30.4% improvement for diabetic MAFLD (0.519 vs 0.398) over the respective best-performing benchmark. The results underscore the clinical value and utility of integrating clinical and survey-based data in the prediction of MAFLD phenotypes among adults.

**Conclusions:**

The proposed method is a viable approach for MAFLD phenotype prediction. It is more effective in identifying at-risk adults than many prevalent data-driven analytics methods and thereby can enhance clinical decision-making and support patient-centric care and management.

## Introduction

### Background

Metabolic dysfunction–associated fatty liver disease (MAFLD) is a leading cause of chronic liver disease, affecting more than one-third of the global population [1,2] and resulting in annual, direct medical costs of US $103 billion in the United States and €35 billion (US $40 billion) in Europe [3]. The relabeling of nonalcoholic fatty liver disease as MAFLD reflects a deeper understanding of fatty liver disease [4]. It also helps identify adults at risk of serious prognoses [5] such as liver cirrhosis and hepatocellular carcinoma, which account for most liver-related deaths [6,7]. The exacerbation of comorbid conditions due to MAFLD amplifies its clinical significance; patients with chronic liver diseases often develop severe infections, chronic cardiovascular or kidney disease, cancer, and death [8]. Yet, therapeutic options for devastating MAFLD-induced liver diseases are limited. Liver transplantation is the optimal treatment [9,10] but is greatly restricted by organ availability and financial costs [11].

A diagnosis of MAFLD requires hepatic steatosis in the presence of excessive weight, type 2 diabetes mellitus, or metabolic dysregulation, manifested in the obese, diabetic, and lean phenotypes (subtypes) of MAFLD, respectively [12]. These phenotypes have distinct prognostic values [5], fibrotic burden [13,14], and complications [15,16]. For example, the diabetic phenotype is characterized by severe insulin resistance and is associated with the highest risk of any-cause and disease-specific mortality [17]. The obese phenotype is related to lifestyle factors (eg, diet and physical inactivity) and can lead to systemic inflammation and metabolic dysfunction. The lean phenotype involves ectopic fat deposition and genetic predispositions to MAFLD, although without obesity [18]. Because of the differences between the MAFLD phenotypes, accurate phenotype prediction is crucial for clinical decision-making, personalized care planning, and efficient resource allocation [19]. With relevant insights into the underlying etiology and pathology [20], effective phenotype prediction can facilitate patient stratification and treatment planning for streamlining diagnostic procedures, optimizing the use of laboratory tests or imaging, and specifying necessary lifestyle changes, all of which have cost-containment implications [21-23].

Physicians usually rely on liver biopsies [24,25] or score-based methods [26,27] that require contemporaneous clinical data, impose substantial costs, and misidentify at-risk adults. These constraints favor the potential of data-driven analytics for supporting timely identification of at-risk adults such that clinicians can formulate actionable risk reduction measures and effective patient stratification and researchers can design more appropriate clinical trials and treatment plans [28]. Despite the promise, data-driven analytics for MAFLD phenotyping face several challenges. First, MAFLD is a multisystem disease [29] because clinical, family genetics, lifestyle, and socioeconomic factors can influence fatty liver development and progression [30]. Incorporating such heterogeneous data in analytics methods, which typically are gathered from different sources, is difficult. For example, surveys designed to gather genetic family history data or lifestyle data tend to have small samples and often suffer from data incompleteness. Second, due to the complex nature of MAFLD, people with the same MAFLD phenotype may exhibit intraphenotype variability in etiology or pathology, which also should be considered for phenotype predictions. Third, both disease classification hierarchy and manifestations of MAFLD involve temporal complexity at the individual level.

### Objective

In an effort to design a data-driven method to predict MAFLD phenotypes more accurately, we developed a novel, 2-stage, contrastive learning–based method. This method leverages graph representation learning, in combination with interindividual similarity, to process integrated individual-level data pertaining to genetic family history or lifestyle, which then can inform downstream predictions by complementing (incomplete) survey-based data with clinical data or vice versa. In addition, the proposed method incorporates multiview, contrastive pretraining that captures intraphenotype variability on the basis of clinical, genetic family history, and lifestyle data. By linking important data from different sources, it constructs individual-level representations for downstream tasks and predictions. Finally, its 2-stage estimation design accounts for disease hierarchy and temporal complexity, such that the proposed method can predict phenotypes among adults more accurately and explicitly than the existing analytics methods.

To demonstrate the predictive efficacy of the proposed method, we used clinical and survey-based data of 4408 adults in Taiwan [31] and included 8 prevalent methods as benchmarks. The results indicated that the proposed method consistently and significantly outperformed all the benchmarks in both *F*_1_-score and area under the curve (AUC). This novel method can predict phenotypes accurately and can potentially contribute to medical informatics research and support personalized care for at-risk adults.

### Related Work

#### MAFLD and Its Phenotypes

Clinically, MAFLD involves metabolic abnormalities [32], and its diagnosis requires hepatic steatosis, which can be determined by imaging, blood biomarker scores, or liver biopsies [20]. Adults diagnosed with MAFLD often differ in their phenotypes, prognoses, and complications [5], leading to distinct clinical manifestations and metabolic characteristics. For example, diabetic MAFLD is characterized by diabetes mellitus, independent of BMI, and exhibits a higher fibrotic burden than other phenotypes, with substantial risks of hepatocellular carcinoma [33] and cardiovascular disease (CVD) [15]. Both obese MAFLD and lean MAFLD are determined on the basis of BMI: ≥23 kg/m² and <23 kg/m², respectively. The former condition involves excess adiposity and is associated with insulin resistance, systemic inflammation, and increased risk of cardiovascular complications [34]. The latter, also known as metabolic dysregulation, is characterized by metabolic abnormalities, and individuals with this phenotype are at a greater risk of liver-related complications and mortality [21]. Because both obese MAFLD and lean MAFLD are determined on the basis of BMI, they can be considered in combination for phenotype predictions. Phenotypic heterogeneity reflects the significant complexity of MAFLD and its varied pathophysiological mechanisms [35], which stem from demographic characteristics, clinical variables, lifestyle factors, and genetic predisposition [36].

In turn, the heterogeneity and complexity of MAFLD make timely, accurate phenotype prediction important but difficult. Notably, MAFLD is reversible in its early stages, with appropriate lifestyle changes and clinical interventions [35]. On the other hand, advanced stages can induce liver diseases and are associated with poor prognoses [37]. In general, accurate phenotype predictions are needed within a 1-year timeframe [38] because MAFLD often exhibits few or no directly observable symptoms until liver damage has occurred. By identifying at-risk adults in a timely manner, physicians can encourage lifestyle changes such as dietary alterations or reduced alcohol consumption [39,40] and plan for laboratory tests or imaging examinations (eg, abdominal ultrasound) [36].

#### Data-Driven Analytics Methods for Patient Risk and Outcome Predictions

Existing data-driven analytics for MAFLD phenotype predictions rely on regression-based [41-43], tree-based [44-46], neural network (NN)–based [47-49], or graph-based [50-52] methods. Regression-based methods, such as Cox regression–based risk estimation [42] and logistic regression models [43], use statistical modeling to predict patient risk and outcomes, support patient risk predictions, and identify important factors. However, these methods cannot deal with high-dimensional data or nonlinear relationships and often make strong data property assumptions. A tree-based method can model nonlinear relationships and derive predictions by applying variable values to split the data recursively, as exemplified by decision tree (DT) [44], random forest (RF) [45], and extreme gradient boosting (XGBoost) [46] methods. While intuitive and interpretable, tree-based methods struggle with overfitting in the presence of noise or data sparsity, and they cannot handle missing data or individual heterogeneity effectively [53]. The deep learning, NN-based methods are able to model complex relationships and nonlinear interactions [54]. For example, deep autoencoders [49] and multilayer perceptron (MLP) [48] methods are advantageous for representing multisource data with high-dimensional features. But they can be difficult to train and are prone to overfitting, especially with insufficient, incomplete, or low-quality data [55]. Finally, graph-based methods represent data as nodes and edges in a graph; they are designed to capture complex relationships and interactions among entities (eg, patients and medications) to inform downstream predictions. Representative methods include graph convolutional networks (GCNs) [56], graph attention networks (GATs) [57], and GraphSAGE [58]. Despite their general effectiveness, graph-based methods rely on predefined graph structures, which can restrict their ability to account for complex, multifaceted, individual feature interactions.

As summarized in Table 1, the existing analytics methods seem generally effective for estimating patient risk and outcome, but their direct use for MAFLD phenotype prediction is insufficient for several reasons. First, many methods depend on clinical data available in electronic health records, which prevents them from accounting for the multifactorial nature of MAFLD. For example, effective phenotype prediction needs to consider genetic family history and lifestyle data, but the incorporation of such data complicates the modeling and obscures patterns essential for accurate prediction, in addition to sample size and data incompleteness issues. Second, most of the prevalent methods do not capture intraphenotype variability, which is critical for downstream predictions. For example, semisupervised (eg, contrastive) learning can deal with complex representations [59-61], but its use requires data augmentation [62-65] and complementary views [66], in addition to the tabular data common in healthcare settings. Third, MAFLD phenotype prediction involves disease classification hierarchy and temporal dynamics. For instance, individuals are classified as those with and without MAFLD (MAFLD and non-MAFLD, respectively), and those with MAFLD need to be further classified into distinct phenotypes by a selective layer, which implies a priori knowledge to inform appropriate feature selection.

**Table 1.** Comparison of this study with representative previous studies.

Study	Method	Multisource data integration	Data heterogeneity	Intraphenotype variability	Disease dynamics
Jia et al (2019) [42]	Regression-based	No	No	No	No
Yang et al (2024) [67]	Regression-based	Yes	No	No	No
Książek et al (2021) [43]	Regression-based	No	No	No	No
Pasadana et al (2021) [68]	Tree-based	No	No	No	No
Wang et al (2019) [69]	Tree-based	No	No	No	Yes
Hashem et al (2012) [70]	NN-based^a^	No	No	No	Yes
Franco et al (2021) [49]	NN-based	Yes	No	No	No
Chowdhury et al (2024) [51]	Graph-based	No	No	Yes	No
Zhang et al (2022) [52]	Graph-based	Yes	No	No	No
Zheng et al (2022) [71]	Graph-based	Yes	No	No	Yes
This study	2-Stage, contrastive learning–based	Yes	Yes	Yes	Yes

a NN: neural network.

## Methods

### Materials

We used 2-year longitudinal data of 4408 adults, obtained from a major healthcare organization in Taiwan, to evaluate the proposed method in comparison with 8 prevalent methods. No adults in the sample had MAFLD in year 1. For each person, the data include 2 demographic variables, 36 clinical variables, 32 lifestyle variables, and 42 genetic family history–related variables. Multimedia Appendix 1 provides the description and coding of variables. With these data, we evaluated the ability of each method to predict whether a person would develop MAFLD in year 2 and, if so, of which phenotype.

Of the 4408 individuals in our sample, 2999 (68.1%) were women, and 1409 (31.9%) were men, with an average age of 58.18 (SD 12.94) years. The outcome class distribution was imbalanced: 85.0% non-MAFLD (3747/4408), 11.5% nondiabetic MAFLD (507/4408), and 3.5% diabetic MAFLD (154/4408). We used class weights during model training to address the imbalance issue. Prior to making phenotype predictions, we applied *z* score standardization to numeric variables and one-hot encoding to categorical variables to prepare the data.

### Ethical Considerations

This study was approved by the Chang Gung Medical Foundation Institutional Review Board (201800270B0). All procedures were performed in accordance with relevant guidelines and regulations. Written informed consent was obtained from all participants. All patient information was anonymized prior to analysis, and the study complied with ethical standards for research involving deidentified healthcare data. Participants were informed that their involvement was voluntary and that they could withdraw from the study at any time without penalty. No financial compensation was provided.

### Proposed Method

#### Problem Definition

Let D be individual demographics, C represent clinical variables, S denote genetic family history–related and lifestyle data, and Y indicate distinct MAFLD outcomes. Phenotype prediction represents a multiclass classification task: given D, C, and S, the objective is to effectively process S based on the observed values, then integrate with D and C to predict whether an individual is likely to develop a specific MAFLD phenotype within a 1-year timeframe. By effectively processing S, it is possible to extract useful information from S, to better cope with the missingness that often arises among self-reported genetic family history data and lifestyle data for improved predictive efficacy. We considered 3 outcome classes for the multiclass classification task, $Y=\left\{Y_{1},Y_{2},Y_{3}\right\}$, which correspond to the non-MAFLD, nondiabetic MAFLD, and diabetic MAFLD phenotypes, respectively. The combination of obese MAFLD and lean MAFLD phenotypes into a single outcome class (nondiabetic MAFLD) is justified because both phenotypes rely solely on BMI. It also simplifies the outcome class classification and allows for meaningful, accurate predictions, in that physicians can readily separate obese and lean MAFLD according to BMI values, which offers clinical practicality [33,72] and facilitates predictions [73,74].

#### Architectural Framework

**Figure 1.**
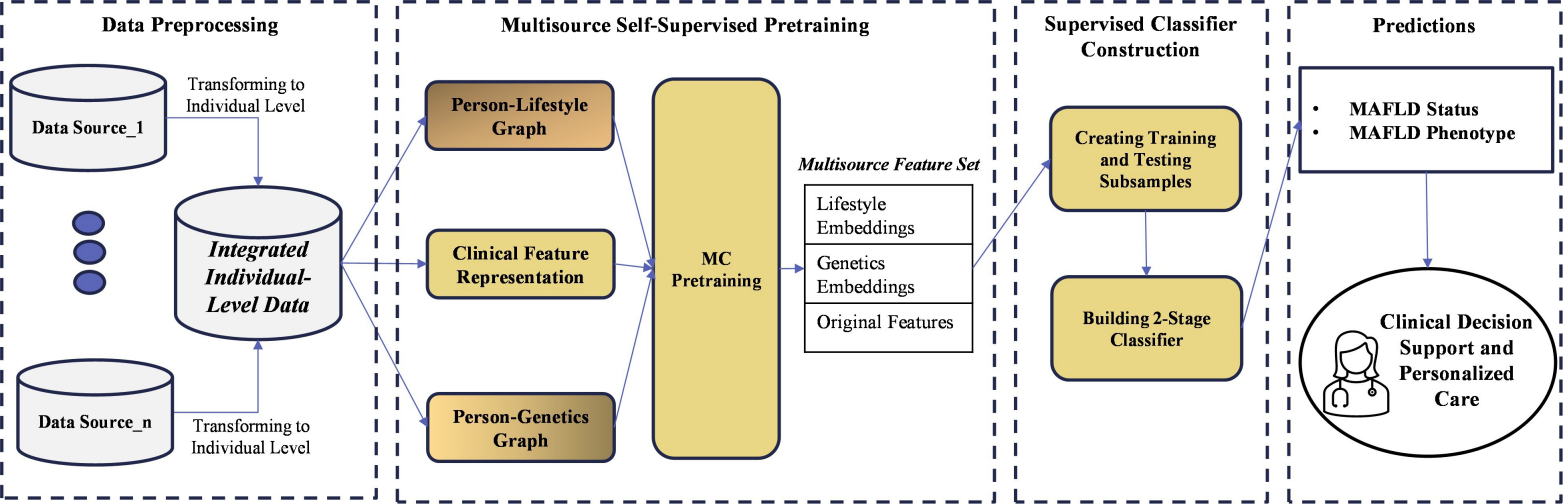
Architectural framework of the proposed method. MAFLD: metabolic dysfunction–associated fatty liver disease; MC: multiview contrastive.

Figure 1 depicts the proposed method’s architectural framework and highlights its 3 important components: graph representation learning, multiview contrastive pretraining, and 2-stage risk estimation. With graph representation learning, the method uses sparse, incomplete survey data to build 2 individual-feature bipartite networks, a person-lifestyle graph and a person-genetics graph, which are used to learn graph representations. The multiview contrastive pretraining component then uses the individual graph representations as inputs to capture intraphenotype variability and create lifestyle and genetics embeddings. Finally, these embeddings are combined with demographic and clinical data in the 2-stage risk estimation process to predict the likelihood of each outcome class for an individual.

#### Graph Representation Learning

We used lifestyle and genetic family history data to perform the novel graph representation learning and construct both person-lifestyle and person-genetics networks. The former captures relationships among individuals according to their lifestyle predispositions (eg, shared dietary habits and physical activities). The latter leverages genetic family history–related variables (eg, shared alleles and single nucleotide polymorphisms) that can influence individuals’ biological or genetic predispositions. These 2 networks were constructed separately to enable the graph representation learning component to concentrate on unique structures and relationships intrinsic to each type of data, thereby capturing the interplay of lifestyle and family genetic variables.

**Figure 2.**
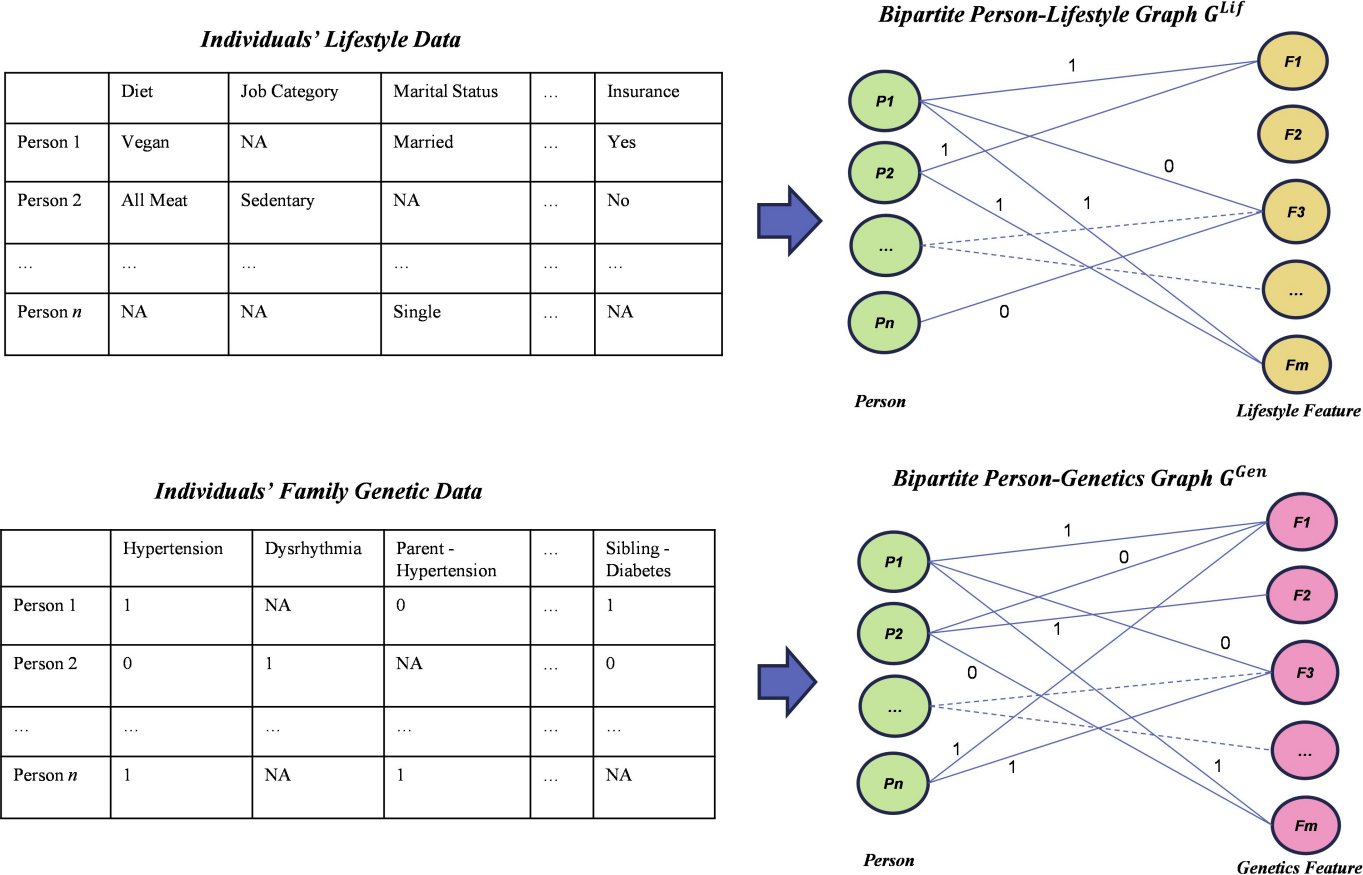
Graph representation learning component of the proposed method. NA: not applicable.

Figure 2 illustrates the construction of 2 bipartite networks. For the person-lifestyle bipartite network, $G^{Lif}=\{V_{P}^{Lif},V_{F}^{Lif},E^{Lif}\}$, $V_{P}^{Lif}=\{P_{1},P_{2},\ldots ,P_{N}\}$ refers to a set of individuals, $V_{F}^{Lif}=\{F_{11},F_{12},\ldots F_{ij},\ldots ,F_{MJ}\}$ represents lifestyle features, and $E^{Lif}$ denotes an edge set that links $V_{P}^{Lif}$ and $V_{F}^{Lif}$. N and M denote the total number of individuals and lifestyle feature values, respectively. For each lifestyle feature, multiple nodes are used to indicate its plausible (coded) values. J denotes the number of distinct values or categories of $F_{M}$; thus, $F_{ij}$ denotes the jth category of feature $F_{i}$. If person $P_{u}$ has a value on lifestyle feature $F_{v}$ of the jth category, there exists an undirected link $e_{u,vj}$ between nodes $P_{u}$ and $F_{vj}$, and the edge weight reflects $P_{u}$’s value on feature $F_{vj}$.

For the person-lifestyle bipartite network, we used GraphSAGE [58] to learn representations for the nodes and edges. We relied on triplet loss to train the graph representation model, which involved an anchor node, a positive sample (neighboring nodes or the node itself if no neighbors existed), and a negative sample:


(1) L=max(0,d(f(a),f(p)−f(a),f(n))+α)

where a is the anchor node, p is the positive node, n is the negative sample, d(∙) is the distance function, f(∙) is the embedding function, and α is a margin parameter. $A_{P_{i}}^{Lif}$ represents the learned node embedding for each person $P_{i}$. Similarly, we built the person-genetics bipartite network, $G^{Gen}=\{V_{P}^{Gen},V_{F}^{Gen},E^{Gen}\}$, to learn the genetic representation $A_{P_{i}}^{Gen}$. The representations learned from these 2 networks provided the input for the contrastive pretraining component.

#### Multiview Contrastive Pretraining

Originally developed for computer vision tasks, contrastive learning leverages data augmentation and complementary views for effective representation learning [66]. Conventional, supervised learning faces multifaceted challenges, especially when dealing with high intraclass variance and imbalanced outcome class distribution. Contrastive learning offers a viable solution by learning data representations through instance discrimination. The core idea is intuitive: instead of solely relying on labeled examples, contrastive learning learns to distinguish among different patients while ensuring that similar patients have similar representations in the learned feature space. This self-supervised approach can learn robust features, particularly in scenarios involving limited or imbalanced labeled data. However, existing contrastive learning methods, such as MoCo [63] and SimCLR [65], rely heavily on data augmentation techniques such as cropping and rotation in images, which are not directly applicable to structured patient data.

We designed a novel multiview contrastive pretraining component that leverages multiple context-specific representations to capture intraphenotype variability. In the proposed method, multiview contrastive learning examines patients’ clinical profiles from multiple perspectives and learns discriminative representations that better predict infrequent but important MAFLD subtypes while maintaining performance across different categories. For this task, an intuitive learning objective can be defined by the cosine similarity among individuals, according to the person-lifestyle representation $A_{P_{i}}^{Lif}$, person-genetic representation $A_{P_{i}}^{Gen}$, and clinical data C. The intent is to capture intraphenotype variability. We applied guided, collaborative training to steer the training process, for which we used clinical variables for the teacher view and survey-based, context-specific representations ($A_{P_{i}}^{Lif}$ and $A_{P_{i}}^{Gen}$) for the learner views. The resulting model can integrate and align critical information from clinical and survey-based data.

**Figure 3.**
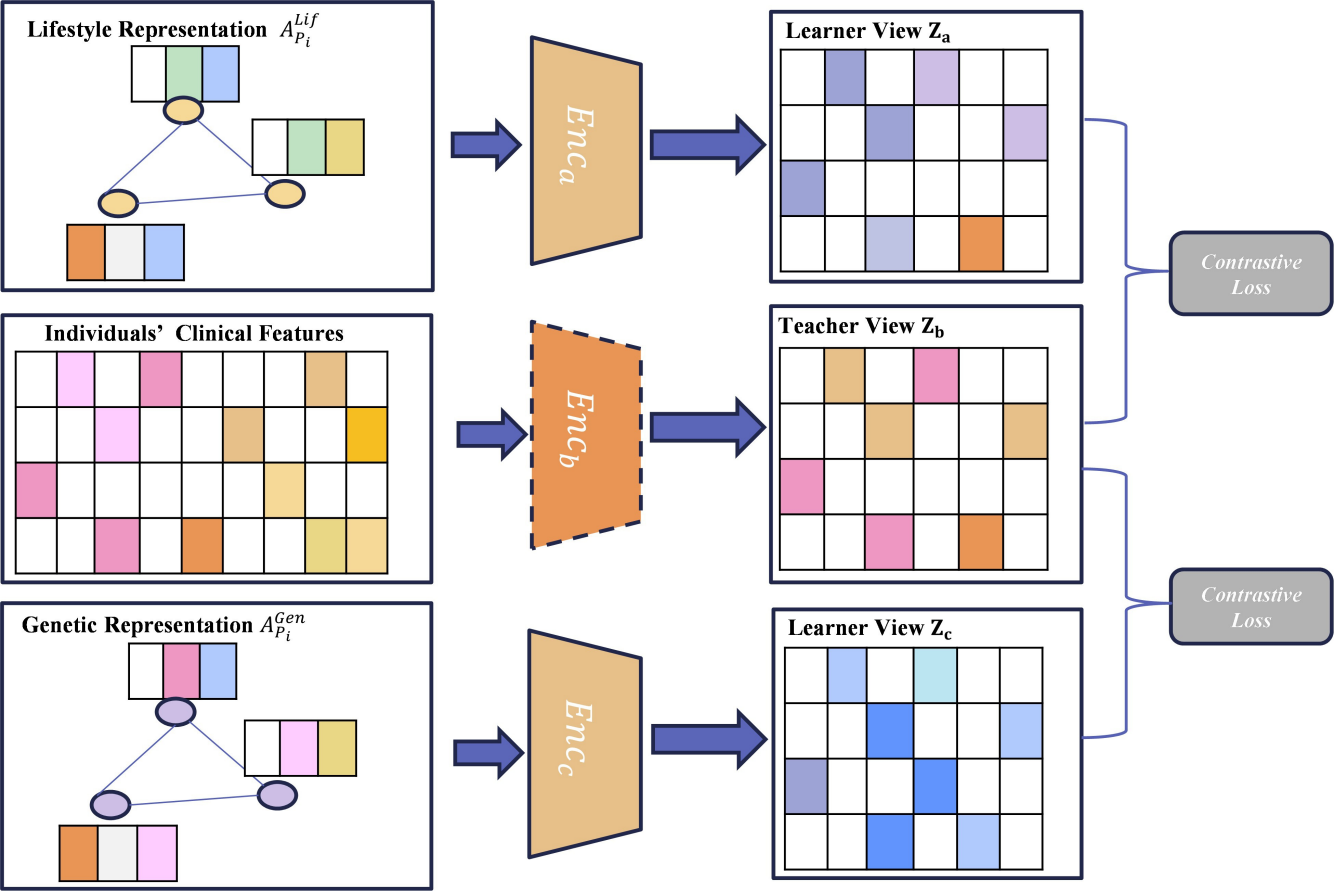
Multiview contrastive learning component of the proposed method.

Figure 3 depicts the contrastive pretraining component, in which 3 encoders (${Enc}_{a}$, ${Enc}_{b}$, and ${Enc}_{c}$) process the representations of lifestyle data, clinical data, and genetic family history data, respectively. Thus, ${Enc}_{b}$ is pretrained with an autoencoder to produce the teacher view that anchors the learning process. As learner views, ${Enc}_{a}$ and ${Enc}_{c}$ are trained according to ${Enc}_{b}$ during the contrastive learning process. Both ${Enc}_{a}$ and ${Enc}_{c}$ adopt the same 3-layer MLP with nonlinear activation functions. The outputs of ${Enc}_{a}$, ${Enc}_{b}$, and ${Enc}_{c}$ are represented by $z_{a}$, $z_{b}$, and $z_{c}$, which denote the embeddings of lifestyle, clinical, and genetic family history data, respectively. For a person $P_{i}$, the objective is to align the cosine similarity of the embeddings of positive pairs {$z_{a}^{\left(i\right)},z_{b}^{\left(i\right)}$} and {$z_{c}^{\left(i\right)},z_{b}^{\left(i\right)}$}, according to the infoNCEloss:


(2) $$L_{contrastive}(z_{a}^{\left(i\right)},z_{b}^{\left(i\right)})=-\log\frac{\exp(\text{sim}(z_{a}^{\left(i\right)},z_{b}^{\left(i\right)})/\tau_{batch})}{\sum_{k=1}^{n}\exp(\text{sim}(z_{a}^{\left(i\right)},z_{b}^{\left(k\right)})/\tau_{batch})}$$

where sim(⋅,⋅) is the similarity function, and $\tau_{batch}$ is the temperature parameter.

In contrastive learning, fixed temperature settings are generally ineffective for heterogeneous data distributions [75]. Therefore, we designed an adaptive temperature network (ATN) to adjust the temperature, $\tau_{batch}$, dynamically. As a lightweight NN, the ATN uses batch-level aggregated statistics as input and generates a single temperature value:


(3) $$V_{batch}=\frac{1}{n}\sum_{i}^{n}z_{b}^{\left(i\right)}$$

and


(4) $$\tau_{batch}=W\cdot Relu\left(V_{batch}\right)$$

where n is the batch size; $V_{batch}$ is the aggregated feature representation, calculated as the batch average of clinical representations $\left\{Z_{b}^{\left(i\right)}\right\}$; and $\tau_{batch}$ is the temperature value for each data batch.

Both ${Enc}_{a}$ and ${Enc}_{c}$ are trained with a cross-entropy loss


(5) $$\begin{array}{l} L_{total}=L_{contrastive}\left(z_{a},z_{b}\right)+L_{contrastive}\left(z_{c},z_{b}\right) \end{array}$$

where $L_{contrastive}\left(z_{a},z_{b}\right)$ and $L_{contrastive}\left(z_{c},z_{b}\right)$ reflect the contrastive loss between $z_{a}$ and $z_{b}$ and $z_{b}$ and $z_{c}$, respectively. Multiview contrastive learning ensures that the learned lifestyle and family genetics embeddings (learner view) align with the clinical embeddings (teacher view), which enhances representation quality.

#### Two-Stage Risk Estimation

**Figure 4.**
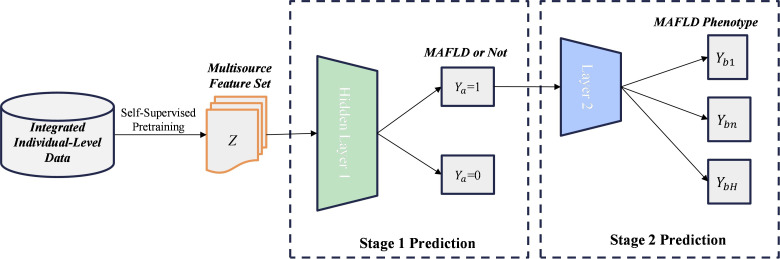
Two-stage phenotype prediction component of the proposed method, where Y_a_ represents the first-stage binary prediction, indicating the presence (Y_a_=1) or absence (Y_a_=0) of any MAFLD phenotype. Y_b_ represents the second-stage probability distribution over the H specific phenotypes, which is subsequently estimated if Y_a_=1. Y_b1_, Y_bn_, and Y_bH_ denote the estimated probabilities for the first, -th, and -th (final) specific MAFLD phenotypes, respectively. MAFLD: metabolic dysfunction–associated fatty liver disease.

Finally, the 2-stage deep NN component for MAFLD phenotype prediction targets important interphenotype relationships. As depicted in Figure 4, this component estimates whether a person is likely to develop MAFLD (${\hat{Y}}_{i,a}=\left[{\hat{Y}}_{i,a}^{1},{\hat{Y}}_{i,a}^{2}\right]$), such that ${\hat{Y}}_{i,a}^{1}=1$ if there is an indication of any MAFLD phenotype and ${\hat{Y}}_{i,a}^{1}=0$ otherwise. In the former case, the component then estimates the likelihood of a specific phenotype and produces the probability distribution ${\hat{Y}}_{i,b}=\left[{\hat{Y}}_{i,b}^{1},\ldots ,{\hat{Y}}_{i,b}^{H}\right]$, corresponding to distinct phenotypes, where H is the total number of phenotypes. This hierarchical estimation design enables the proposed method to capture general characteristics of MAFLD and distinct phenotypes for predictions. The overall probability distribution ${\hat{Y}}_{i}$ can be calculated as follows:


(6) $${\hat{Y}}_{i}=[1-{\hat{Y}}_{i,a}^{1},\;{\hat{Y}}_{i,b}^{1}\cdot {\hat{Y}}_{i,a}^{1},\;{\hat{Y}}_{i,b}^{2}\cdot {\hat{Y}}_{i,a}^{1},\;{\hat{Y}}_{i,b}^{3}\cdot {\hat{Y}}_{i,a}^{1},\;\ldots ,\;{\hat{Y}}_{i,b}^{H}\cdot {\hat{Y}}_{i,a}^{1}]$$

In the 2-stage estimation process, we also designed a loss function to train the proposed method:


(7) $$\begin{array}{l} L_{\text{total}}=-\sum_{i=1}^{N}y_{i}^{\left(n\right)}\log\;\left({\hat{y}}_{i}^{\left(n\right)}\right)+\\ \gamma \cdot \left(-\sum_{i=1}^{K}y_{i,a}^{\left(k\right)}\log\;\left({\hat{y}}_{i,a}^{\left(k\right)}\right)\right)\\ +\lambda \cdot \left(-\sum_{i=1}^{M}y_{i,b}^{\left(m\right)}\log\;\left({\hat{y}}_{i,b}^{\left(m\right)}\right)\right) \end{array}$$

The first term of $L_{total}$ is the negative log-likelihood loss, calculated according to the actual and predicted MAFLD phenotype. The second and third terms denote the losses in the first and second stages, respectively, and γ and are hyperparameters that control the trade-offs among these 3 terms. Specifically, ${\hat{y}}_{i}^{\left(n\right)}$ indicates the overall predicted probability of the nth class for person i, ${\hat{y}}_{i,a}^{\left(k\right)}$ is the estimated probability of MAFLD (binary, k=2), and ${\hat{y}}_{i,b}^{\left(m\right)}$ denotes the estimated probability of the mth phenotype for individuals predicted to have MAFLD in stage 2. With $L_{total}$, our method learns interphenotype relationships for phenotype prediction.

### Evaluations

Eight prevalent methods were included as benchmarks: DT [44], RF [45], XGBoost [46], MLP [48], autoencoder [49], GAT [57], GCN [56], and GraphSAGE [58]. These methods represent different analytics approaches and are frequently used for clinical prediction tasks; therefore, they are suitable for performance comparisons. Many of these benchmark methods are not designed to deal with incomplete data. Because the sample had missing values, we applied k-nearest neighbor (k=5) imputation [76-78] to the dataset and used one-hot encoding for categorical variables during data preprocessing to ensure consistency and comparability in the evaluations, that is, all methods used the same preprocessed data for fair comparisons. The only difference was that the proposed method also used the raw, nonimputed survey data (genetic family history and lifestyle data) as input for graph representation learning and contrastive learning, which are components capable of handling missing values. Moreover, we conducted an ablation study to examine the relative contribution of each key component to the proposed method’s overall performance.

To examine the prediction performance of each method, we randomly split the sample 10 times, using different random seeds to ensure robustness. In each trial, we used 80% of the data for model training and the remaining 20% for testing [76]. We also conducted 5-fold cross-validation on the training data prior to the evaluations and performed a series of analyses to fine-tune the key parameters of each method. Multimedia Appendix 2 summarizes important parameter values of the respective methods. Performance assessments relied on precision, recall, *F*_1_-score, and AUC values. We did not consider accuracy, as it could not reflect prediction performance due to the imbalanced distribution of the outcome classes [79]. Compared with precision or recall, the *F*_1_-score and AUC are arguably better indicators of a method’s efficacy of predicting MAFLD phenotypes. As reported by Docherty et al [80], we adopted a one-versus-rest strategy to assess each outcome class and compared the respective AUC values of all methods, which supports a fair, holistic analysis of their ability to predict MAFLD phenotypes.

## Results

### Overall Prediction Performance

Table 2 presents each method’s prediction performance across 10 trials. The proposed method has a 2-stage estimation design—stage 1 estimates whether an individual will develop MAFLD, and stage 2 predicts the likelihood of each MAFLD phenotype. Therefore, we report the results for each stage separately. As Table 2 shows, the proposed method attained higher AUC values in both stages, indicating its ability to distinguish patients with different outcomes. In stage 1, it accurately identified adults likely to develop MAFLD, with few false alarms, as signified by the relatively high precision and recall values. In stage 2, the proposed method generated effective predictions by consolidating the stage 1 results. The multiclass prediction results in stage 2 also allowed for direct comparisons with the benchmark methods. As seen in Table 2, the proposed method outperformed all benchmarks on both *F*_1_-score and AUC. It exhibited a 7.2% improvement in AUC over the best-performing benchmark (0.898 vs 0.838) and had a 16.6% higher *F*_1_-score than the best-performing benchmark (0.652 vs 0.559). Paired two-tailed *t* tests performed to examine differences in AUC indicated that the observed improvements were statistically significant (*P*<.001).

**Figure 5.**
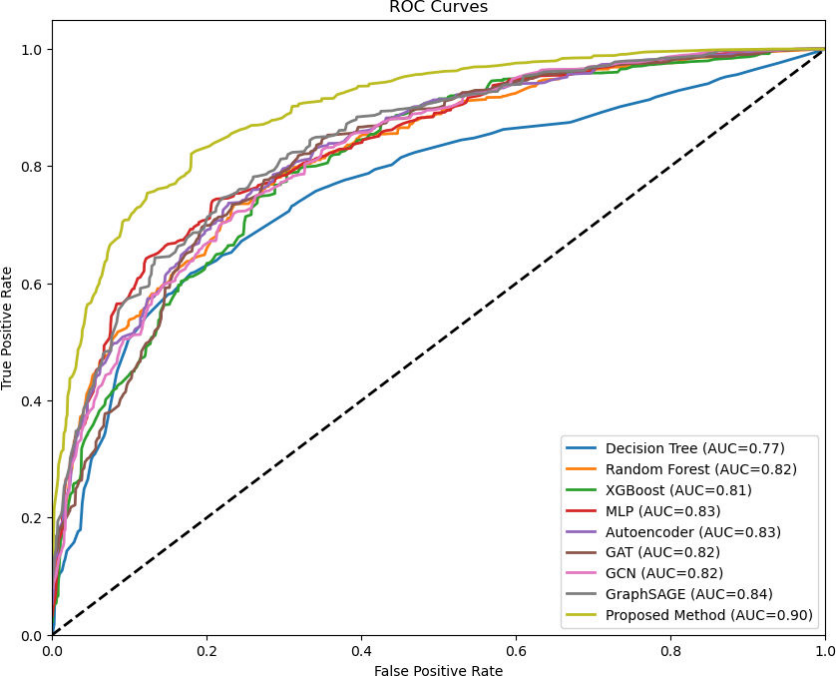
Area under the curve (AUC) values for the investigated methods. GAT: graph attention network; GCN: graph convolutional network; MLP: multilayer perceptron; ROC: receiver operating characteristic.

Figure 5 presents the respective receiver operating characteristic curves of all methods. The proposed method’s AUC curve was notably better than that of any benchmark method. This result further affirms its superior efficacy in estimating MAFLD phenotypes among adults compared with many prevalent methods.

**Table 2.** Overall performance of each investigated method.

Method	Performance metric, mean (SE)			
	Precision	Recall	*F*_1_-score	AUC^a^
DT^b^	0.549 (0.012)	0.468 (0.007)	0.493 (0.007)	0.765 (0.007)
RF^c^	0.576 (0.021)	0.542 (0.019)	0.541 (0.016)	0.819 (0.007)
XGBoost^d^	0.598 (0.019)	0.490 (0.015)	0.525 (0.019)	0.812 (0.019)
MLP^e^	0.567 (0.008)	0.570 (0.019)	0.557 (0.008)	0.831 (0.002)
Autoencoder	0.537 (0.011)	0.566 (0.023)	0.528 (0.010)	0.832 (0.003)
GAT^f^	0.528 (0.014)	0.542 (0.022)	0.512 (0.010)	0.823 (0.004)
GCN^g^	0.505 (0.011)	0.554 (0.012)	0.512 (0.014)	0.824 (0.005)
GraphSAGE	0.540 (0.010)	0.598 (0.011)	0.559 (0.009)	0.838 (0.004)
Proposed method (stage 1)	0.713 (0.016)	0.745 (0.008)	0.726 (0.011)	0.859 (0.004)
Proposed method (stage 2)	0.644 (0.022)	0.678 (0.027)	0.652 (0.013)	0.898 (0.003)

a AUC: area under the curve.

b DT: decision tree.

c RF: random forest.

d XGBoost: extreme gradient boosting.

e MLP: multilayer perceptron.

f GAT: graph attention network.

g GCN: graph convolutional network.

### Prediction Performance for Each Outcome Class

In addition to overall performance, we examined the respective methods’ performance for each outcome class. As shown in Table 3, the proposed method achieved the highest *F*_1_-score and AUC values for each outcome class, reaffirming its superior prediction ability. It attained a higher *F*_1_-score (0.913) and AUC (0.859) for non-MAFLD than the respective best-performing benchmarks (DT: *F*_1_-score=0.908; GraphSAGE: AUC=0.801). The performance improvements were especially prominent for the MAFLD phenotypes. For nondiabetic MAFLD, our method achieved an *F*_1_-score of 0.531, much higher than that of the best-performing benchmark (MLP: *F*_1_-score=0.400), exhibiting a 32.8% improvement. It also attained the highest AUC (0.878), higher than that of the best-performing benchmark (GraphSAGE: AUC=0.804). For diabetic MAFLD, the proposed method’s *F*_1_-score (0.519) was 30.4% higher than that of the best-performing benchmark (GraphSAGE: *F*_1_-score=0.398). Moreover, its precision value was superior to that of other methods, suggesting that it can identify adults who are likely to develop diabetic MAFLD with fewer false alarms.

**Table 3.** Prediction performance of each method for 3 outcome classes.

Outcome class and method	Performance metric, mean (SE)			
	Precision	Recall	*F*_1_-score	AUC
Non-MAFLD^a^				
Decision tree	0.879 (0.002)	0.941 (0.004)	0.908 (0.002)	0.746 (0.007)
Random forest	0.892 (0.004)	0.938 (0.003)	0.901 (0.004)	0.781 (0.006)
XGBoost^b^	0.881 (0.002)	0.954 (0.003)	0.895 (0.004)	0.798 (0.006)
MLP^c^	0.899 (0.004)	0.898 (0.010)	0.897 (0.003)	0.788 (0.005)
Autoencoder	0.905 (0.004)	0.878 (0.011)	0.892 (0.004)	0.800 (0.005)
GAT^d^	0.899 (0.006)	0.845 (0.023)	0.870 (0.012)	0.777 (0.008)
GCN^e^	0.913 (0.004)	0.825 (0.032)	0.861 (0.018)	0.799 (0.005)
GraphSAGE	0.907 (0.003)	0.875 (0.011)	0.890 (0.005)	0.801 (0.004)
Proposed method	0.925 (0.005)	0.899 (0.017)	0.913 (0.008)	0.859 (0.011)
Nondiabetic MAFLD				
Decision tree	0.436 (0.016)	0.253 (0.021)	0.316 (0.019)	0.781 (0.010)
Random forest	0.444 (0.021)	0.334 (0.031)	0.359 (0.028)	0.787 (0.009)
XGBoost	0.495 (0.020)	0.251 (0.016)	0.329 (0.015)	0.803 (0.005)
MLP	0.423 (0.016)	0.392 (0.026)	0.400 (0.017)	0.800 (0.006)
Autoencoder	0.347 (0.026)	0.344 (0.020)	0.337 (0.014)	0.777 (0.008)
GAT	0.301 (0.022)	0.387 (0.045)	0.323 (0.014)	0.777 (0.007)
GCN	0.280 (0.014)	0.421 (0.055)	0.317 (0.016)	0.765 (0.007)
GraphSAGE	0.384 (0.018)	0.405 (0.023)	0.388 (0.014)	0.804 (0.008)
Proposed method	0.506 (0.016)	0.563 (0.021)	0.531 (0.019)	0.878 (0.003)
Diabetic MAFLD				
Decision tree	0.331 (0.022)	0.210 (0.013)	0.255 (0.015)	0.769 (0.018)
Random forest	0.392 (0.023)	0.381 (0.035)	0.363 (0.024)	0.891 (0.012)
XGBoost	0.450 (0.027)	0.255 (0.010)	0.323 (0.012)	0.848 (0.018)
MLP	0.376 (0.020)	0.421 (0.053)	0.371 (0.020)	0.905 (0.008)
Autoencoder	0.358 (0.045)	0.480 (0.071)	0.354 (0.023)	0.920 (0.003)
GAT	0.378 (0.043)	0.395 (0.061)	0.344 (0.022)	0.915 (0.006)
GCN	0.322 (0.025)	0.417 (0.046)	0.353 (0.025)	0.907 (0.007)
GraphSAGE	0.330 (0.024)	0.519 (0.033)	0.398 (0.023)	0.917 (0.005)
Proposed method	0.500 (0.016)	0.570 (0.042)	0.519 (0.019)	0.957 (0.009)

a MAFLD: metabolic dysfunction–associated fatty liver disease.

b XGBoost: extreme gradient boosting.

c MLP: multilayer perceptron.

d GAT: graph attention network.

e GCN: graph convolutional network.

**Figure 6.**
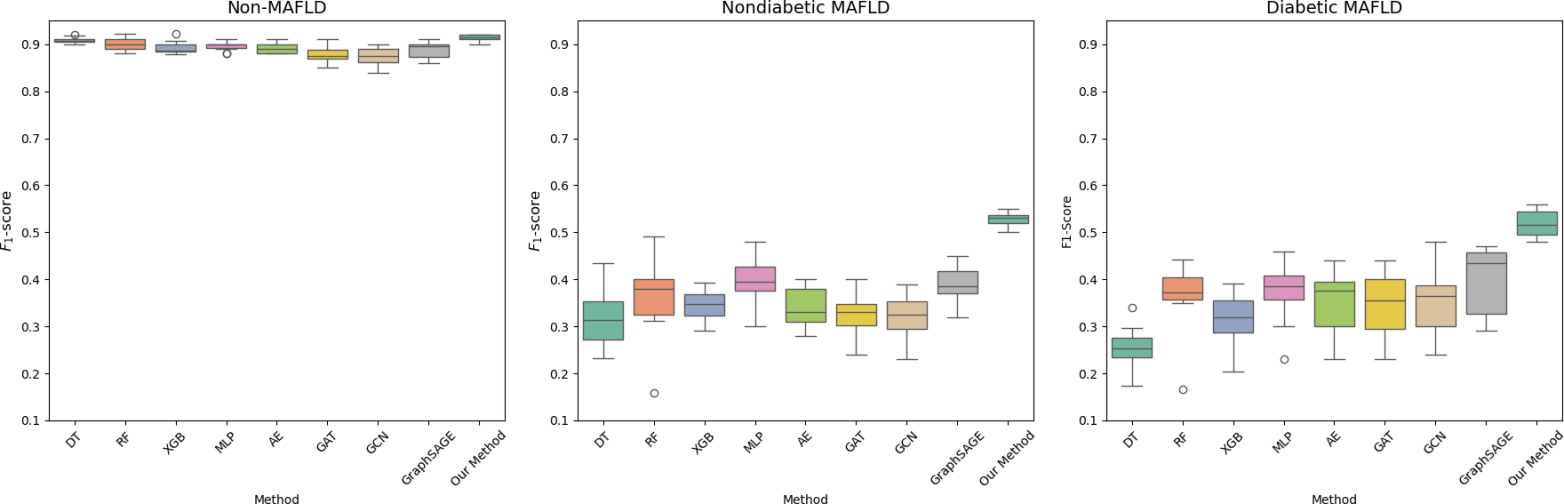
Box plots showing *F*_1_-scores (median and IQR) of each method for different outcome classes. AE: autoencoder; DT: decision tree; GAT: graph attention network; GCN: graph convolutional network; MAFLD: metabolic dysfunction–associated fatty liver disease; MLP: multilayer perceptron; RF: random forest; XGBoost: extreme gradient boosting.

The box plots in Figure 6 indicate the proposed method’s robust performance for each outcome class across 10 trials. It attained high *F*_1_-scores for each outcome class, especially nondiabetic MAFLD and diabetic MAFLD, while the benchmark methods exhibited notably greater variance and occasional outliers. Together, these plots provide further evidence of the proposed method’s efficacy and value for clinical decision-making and patient management.

### Ablation Study

We also performed an ablation study to examine the relative contribution of each key component of the proposed method. We considered MLP, Graph, Graph + contrastive learning, and the (complete) proposed method. In essence, MLP serves as a baseline because it only uses the preprocessed data, without any key components of the proposed method. Graph builds on MLP and includes the graph representation learning of genetic family history and lifestyle data, together with the learned embeddings concatenated to the preprocessed dataset to train the MLP classifier. Graph + contrastive learning further extends Graph by incorporating contrastive learning after graph representation learning. The complete proposed method included all 3 key components. The results of the ablation study (Table 4) revealed how each component contributed to the method’s performance. They jointly produced the best predictions, indicating that MAFLD phenotype prediction can benefit from graph representation, multiview contrastive pretraining, and 2-stage estimation design.

**Table 4.** Results of the ablation study.

Model	AUC^a^
MLP^b^	0.831 (0.002)
Graph	0.847 (0.004)
Graph + CL^c^	0.881 (0.001)
Complete proposed method	0.898 (0.003)

a AUC: area under the curve.

b MLP: multilayer perceptron.

c CL: contrastive learning.

### Interpretability Analysis

**Figure 7.**
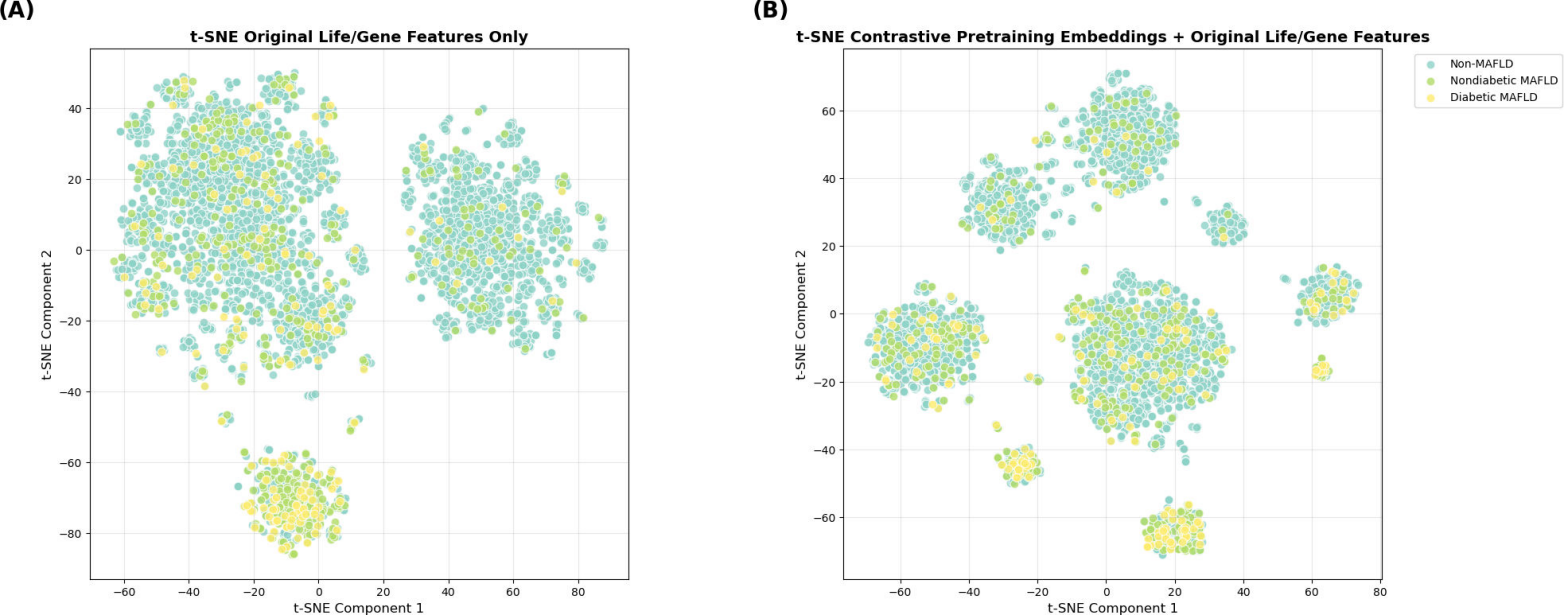
T-distributed stochastic neighbor embedding (t-SNE) visualization of (A) original lifestyle and genetic (life/gene) features and (B) contrastive pretraining embeddings with lifestyle and genetic features. MAFLD: metabolic dysfunction–associated fatty liver disease.

To gain clinical insights into the proposed method’s learned representations, we examined its interpretability by depicting the embeddings visually. Specifically, we applied t-distributed stochastic neighbor embedding (t-SNE) [81] to visualize the contrastive pretraining embeddings and performed a Shapley additive explanation (SHAP) analysis [82] to reveal feature importance. Figure 7A presents a visualization of the original lifestyle and genetic features, and Figure 7B provides a visualization of the features obtained by concatenating the contrastive pretraining embeddings with the original lifestyle and genetic features. The original lifestyle and genetic features exhibited a scattered distribution, without any clear patterns. With contrastive pretraining embeddings, more distinctive clusters emerged, suggesting that patients with similar characteristics tend to cluster more closely than those with dissimilar characteristics. While these visual plots are exploratory without formal proof of class separability, they still illustrate that incorporating contrastive pretraining embeddings can potentially create a more structured, distinguishable representation of patient outcomes for effective MAFLD phenotype prediction.

**Figure 8.**
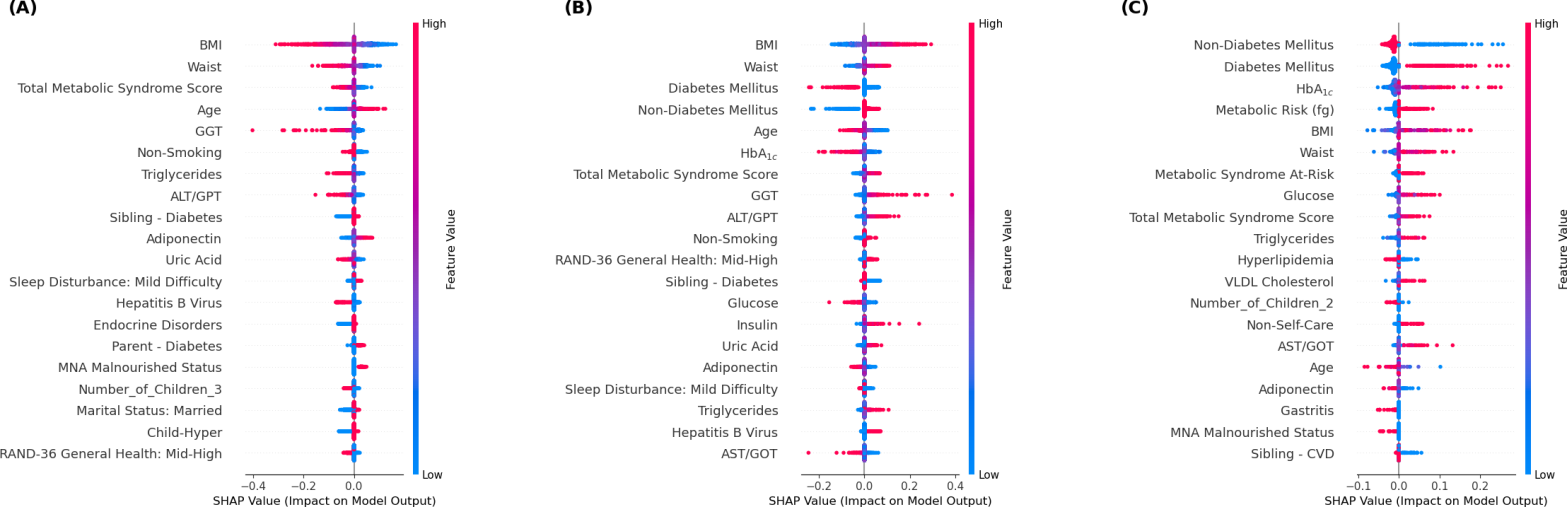
Summary plots of Shapley additive explanation (SHAP) values for (A) non–metabolic dysfunction–associated fatty liver disease (non-MAFLD), (B) nondiabetic MAFLD, and (C) diabetic MAFLD. ALT/GPT: alanine aminotransferase/glutamic-pyruvic transaminase; AST/GOT: aspartate aminotransferase/glutamic-oxaloacetic transaminase; CVD: cardiovascular disease; GGT: gamma-glutamyl transferase; HbA_1c_: hemoglobin A_1c_; MNA: Mini Nutritional Assessment.

We further examined the feature importance for each outcome class, as depicted by the SHAP summary plots in Figure 8. Because the proposed method adopted a 2-stage estimation (architecture) design, the model-agnostic explainer KernelSHAP was used with a background dataset of 100 training instances. For all test instances, SHAP values were computed on a representative model instance (ie, median test AUC across 10 trials). As seen in Figure 8, several metabolic indicators were important predictors consistently across different outcome classes. For example, BMI and waist circumference were highly influential. As Figure 8A shows, high BMI values (marked as red points) greatly reduced the likelihood of non-MAFLD predictions; particularly, high BMI and waist circumference values were associated with a greater likelihood of nondiabetic MAFLD or diabetic MAFLD, as shown in Figure 8B and C. Predictions of nondiabetic MAFLD were influenced by a combination of general metabolic indicators (eg, BMI and waist circumference) and lifestyle factors (eg, smoking and sleep disturbance). For diabetic MAFLD, definitive disease markers and factors related to disease consequence and management, such as self-care status and nutritional status (Mini Nutritional Assessment), appeared to be essential auxiliary predictors. These results align with clinical knowledge and reveal the proposed method’s ability to capture phenotype-specific patterns from patient data, with desirable interpretability.

**Figure 9.**
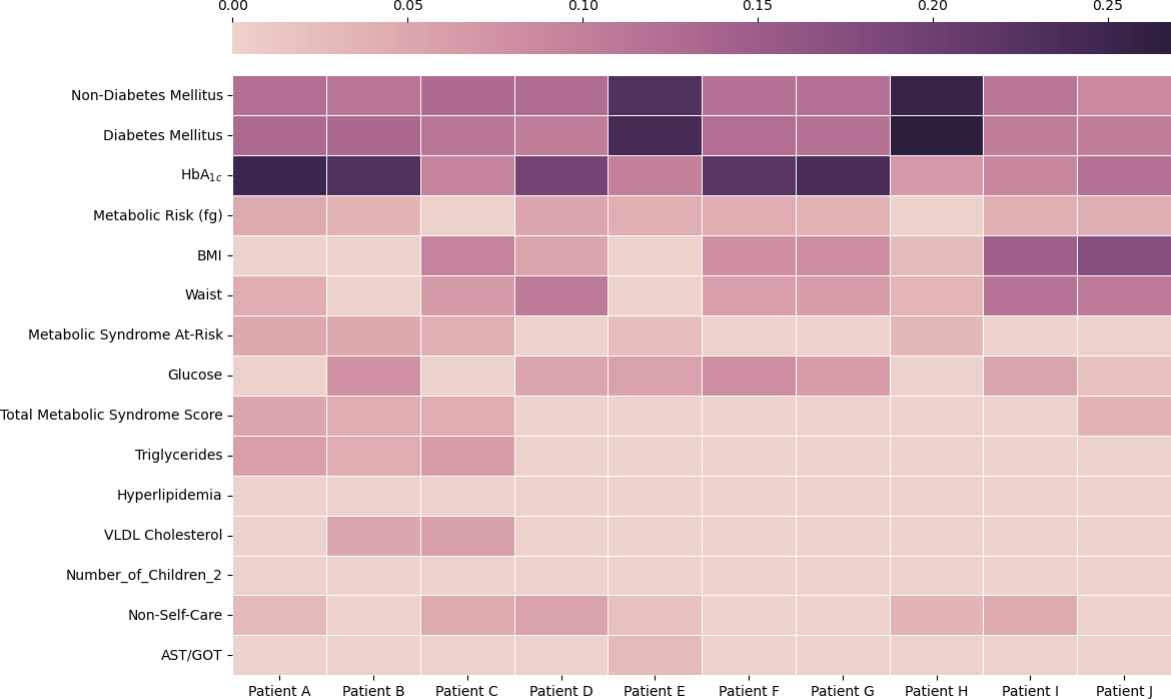
Heat map of sample patients with the predicted phenotype diabetic metabolic dysfunction–associated fatty liver disease (diabetic MAFLD) and top 15 features. AST/GOT: aspartate aminotransferase/glutamic oxaloacetic transaminase; HbA_1c_: hemoglobin A_1c_; VLDL, very-low-density lipoprotein.

Additionally, SHAP analyses allow for reasoning at the individual level. Figure 9 provides a visualization of SHAP values for 10 patients who were predicted to develop diabetic MAFLD. The heat map shows that diabetes mellitus and high hemoglobin A_1c_ diagnoses were consistently important predictors for most patients in this group, including patients B, G, and H. We also observed significant intraphenotype variability among patients. For example, the prediction for patient J was also significantly influenced by BMI and waist circumference, whereas triglycerides were a more important factor for patient C. The interpatient variability can help physicians better understand the impact of different factors at the individual level and thereby support personalized care and treatment planning.

## Discussion

### Principal Findings

The proposed method leverages deep learning to estimate MAFLD phenotypes among adults, using graph representation learning and contrastive learning. It provides several methodological novelties that can advance medical informatics research and enhance clinical decision-making for improved patient management. The evaluation results establish its predictive efficacy, demonstrate the value of combining clinical and survey-based data, and underscore the importance of intraphenotype variability and disease dynamics for MAFLD phenotype prediction. Furthermore, this method is generalizable and can be applied to other prediction tasks in similar clinical scenarios (eg, gauging the risk of diabetes or CVD) that feature multisource data, individual heterogeneities, intraclass variance, and intervariable relationships.

Using the proposed method, physicians will be able to identify individuals at higher risk of fibrosis and generate timely alerts for effective patient-centric care [83], which can mitigate the likelihood of significant disease progression and serious patient outcomes. Accurate prediction of MAFLD phenotypes also helps reduce hepatic complications such as CVD, chronic kidney disease [16], hepatocellular carcinoma [6], osteoporosis, endocrine disorders, and cognitive impairment [84]. The proposed method is capable of distinguishing high-risk versus low-risk adults on the basis of pathogenesis, spanning lifestyle, genetic, and metabolic factors; as a result, the likelihood of fibrosis or cirrhosis can be reduced, with broad implications for precision medicine and drug development [85]. In a related sense, its ability to predict phenotypes in an accurate and timely manner also enables personalized surveillance, treatment choice assessments, lifestyle changes, and treatment planning.

Although the proposed method does not achieve an objectively high *F*_1_-score for MAFLD phenotypes, it still offers meaningful improvements over prevalent methods, even in the presence of the inherent challenges created by highly imbalanced patient clinical data. In our sample, most adults were in the non-MAFLD category, and few had MAFLD phenotypes, which made model training difficult for every method we investigated. This challenge is common to many clinical settings and has been documented across different patient outcome or risk prediction tasks. For example, recent related studies report *F*_1_-scores in the range between 0.10 and 0.51 for minority classes [86,87]. Despite this persistent difficulty, the proposed method consistently outperformed all the benchmarks on MAFLD phenotypes (minority classes), which are clinically important. Hence, the observed improvements with our method represent valuable advances [22,88].

We illustrate the clinical use of the proposed method as a proactive risk stratification approach for clinical decision support and patient management. In stage 1, it estimates the probability of a person developing MAFLD within 1 year. To flag individuals as high risk, a physician can use the probability to select a decision threshold for balancing the trade-off between precision (the proportion of flagged individuals who are truly at high risk) and recall (the proportion of all true positive individuals who are truly flagged as high risk). If the physician prefers high certainty, they can choose a high threshold value. For example, our post hoc analysis showed that by setting the threshold to 0.60, the proposed method’s precision increased to 0.777, that is, approximately 78% of flagged patients indeed developed MAFLD. By choosing an even higher threshold value of 0.70, its precision further increased to 0.820, although at the cost of reduced sensitivity (0.59 in recall). As a result, the physician can identify adults who should be monitored more closely (for example, a semiannual follow-up instead of an annual follow-up), need immediate lifestyle counseling, or require proactive baseline liver function tests to track changes over time.

Furthermore, the proposed method provides additional insights based on the stage 2 estimate, which can support personalized planning and care. In general, obtaining clinically meaningful precision requires a higher threshold value. For example, with a threshold value of 0.50, the proposed method’s precision reached 0.506 for nondiabetic MAFLD and 0.500 for diabetic MAFLD. Emphasizing high-probability instances with a threshold value of 0.70 increased the precision to 0.762 and 0.778, respectively, which would allow physicians to tailor management strategies for adults whose phenotype can be predicted with higher confidence. Additionally, physicians can leverage the instance-level SHAP analysis, as depicted in Figure 9, to identify the specific factors that drive patient risk. While these insights do not directly indicate a definitive diagnosis, they can still guide physicians to engage in preventive care through patient risk stratification, while coping with the challenge of precise phenotype classification. Overall, physicians can adopt an appropriate threshold value to balance precision and recall while minimizing the likelihood of missing at-risk individuals for proactive stratification.

In summary, a multiview architecture leverages complementary information from lifestyle, genetic, and clinical data perspectives for richer representations that help distinguish infrequent yet clinically important MAFLD phenotypes, without sacrificing interpretability. The 2-stage design offers flexibility and additional utility. Accurate and robust estimates in stage 1 help physicians assess whether or not an individual is likely to develop MAFLD for initial screening purposes. In addition to that determination, even a moderate improvement in the *F*_1_-score in stage 2 can facilitate physicians’ decision-making by providing additional information and clinical insights. These valuable risk stratification capabilities enable physicians to identify high-risk adults who may need close monitoring or alternative treatments. The 2-stage design also offers beneficial flexibility. Physicians can adjust their focus across the first or second stage, depending on their objective (eg, early screening, risk stratification, or intervention planning). According to 2 experienced hepatologists (who wish to remain anonymous), “Early, better estimates of individuals’ likelihood of MAFLD is valuable clinically,” and “The use of data-driven analytics methods to predict MAFLD phenotypes can enhance clinical decision-making and personalized patient management” (September 2, 2025). These expert inputs affirm the clinical value and practicality of our proposed method.

### Limitations and Research Directions

This study has several limitations, and it can be extended by further research. First, we used a sample from a single healthcare organization, which offered relatively limited diversity in terms of data sources and patient populations. In a related sense, our sample was imbalanced in the outcome class distribution, which constrained the prediction performance for minority classes, as reflected by the relatively low *F*_1_-scores, which is in line with previous research [87]. Future studies should consider additional data sources and types such as image and text [89] to extend the proposed method, use different patient cohorts to affirm its efficacy, and apply synthetic data augmentation or multimodal foundation models to better address the issue of imbalanced outcome class distribution with cross-modal learning capabilities [62]. Second, because intraphenotype variability introduces complexity with regard to achieving compact clusters in the embedding space, a trade-off arises between variability and compactness, which could restrict the predictive utility for large datasets or different diseases. Therefore, we call for efforts to explore an optimal balance of variability and compactness for both accuracy and generalizability, such as clustering-based contrastive learning [90]. Third, the proposed 2-stage method provides some limited interpretability, through a feature attribution–based approach (ie, SHAP); its contrastive pretraining component deserves further exploration for greater transferability and interpretability. Ongoing efforts could facilitate and interpret embeddings in focal clinical contexts. Fourth, an international, multisociety Delphi process led to the proposal of metabolic dysfunction–associated steatotic liver disease (MASLD) in 2023 [91]. Although our findings might be extrapolated to adults with MASLD [92], the proposed method should be extended with research that tests for differences between MAFLD and MASLD and refines the proposed method to ensure robustness and prediction performance.

### Conclusion

Predicting MAFLD phenotypes among adults is crucial, but existing analytic methods overlook its multisystem nature and phenotypic heterogeneity. As a solution, we developed a novel method that leverages graph representation learning, multiview contrastive pretraining, and a 2-stage estimation design to produce effective predictions that reflect phenotypic heterogeneity, complex relationships, and disease dynamics. It is effective in identifying at-risk adults and thus offers support for clinical decision-making and personalized care. This study reveals a promising pathway to advance health informatics research and clinical practice by leveraging rich, detailed clinical data in electronic health records and survey-based data to predict MAFLD phenotypes.

## Supplementary material

10.2196/75747 Multimedia Appendix 1 Description and coding of variables.

10.2196/75747 Multimedia Appendix 2 Key hyperparameters of the investigated methods.

## Abbreviations

ATN: adaptive temperature network
AUC: area under the curve
CVD: cardiovascular disease
DT: decision tree
GAT: graph attention network
GCN: graph convolutional network
MAFLD: metabolic dysfunction–associated fatty liver disease
MASLD: metabolic dysfunction–associated steatotic liver disease
MLP: multilayer perceptron
NN: neural network
RF: random forest
SHAP: Shapley additive explanation
t-SNE: t-distributed stochastic neighbor embedding
XGBoost: extreme gradient boosting
