# Supplementary material for: Predicting Metabolic Dysfunction–Associated Fatty Liver Disease Phenotypes Among Adults: 2-Stage Contrastive Learning Method
Source: JMIR Med Inform. 2025 Dec 12;13:e75747. doi: 10.2196/75747 (PMC12702840; doi:10.2196/75747)
Supplement: Multimedia Appendix 1 [file medinform-v13-e75747-s001.docx]

**Appendix A: Variable Descriptions and Coding**

| Variable | Type | Description |
| --- | --- | --- |
| *Demographics and Clinical Features* |  |  |
| Patient ID | Character | Patient ID |
| Gender | Binary | Male (1) or Female (0) |
| Age | Integer | Patient age in years |
| Waist | Numeric | Waist circumference in cm |
| Glucose | Numeric | Fasting blood glucose level (mg/dL) |
| Triglycerides | Numeric | Triglyceride level, measured in mg/dL. |
| HDL Cholesterol | Numeric | High-density lipoprotein cholesterol (HDL-C) level, measured in mg/dL. |
| AST/GOT | Numeric | Numeric value of AST (GOT) level in the blood, measured in U/L |
| ALT/GPT | Numeric | Numeric value of ALT (GPT) level in the blood, measured in U/L |
| Gamma GT | Numeric | Gamma-glutamyl transferase (γ-GT), measured in U/L |
| Insulin | Numeric | Fasting insulin level, measured in µU/mL |
| Total cholesterol | Numeric | Total cholesterol level, measured in mg/dL |
| LDL cholesterol level | Numeric | Low-density lipoprotein cholesterol (LDL-C) level (in mg/dL) |
| VLDL Cholesterol | Numeric | Very low-density lipoprotein cholesterol (VLDL-C) level (in mg/dL) |
| Non-HDL Cholesterol | Numeric | Non-HDL cholesterol level, measured in mg/dL |
| Cholesterol to HDL ratio | Numeric | Ratio of total cholesterol to HDL-C, a cardiovascular risk factor |
| LDL to HDL ratio | Numeric | Ratio of LDL-C to HDL-C, a cardiovascular risk factor |
| High-Sensitivity CRP | Numeric | High-sensitivity C-reactive protein (CRP) level (in mg/L) |
| HbA1c | Numeric | Glycated hemoglobin level (HbA1c), specified in a percentage value |
| Uric acid | Numeric | Serum uric acid level, measured in mg/dL |
| Insulin resistance | Numeric | Insulin resistance index |
| Adiponectin | Numeric | Adiponectin level, measured in µg/mL |
| Leptin | Numeric | Leptin level, measured in ng/mL |
| Total vitamin D | Numeric | Total vitamin D level, measured in ng/mL |
| BMI | Numeric | Body mass index (BMI) |
| Diabetes Mellitus | Binary | Indicator for diabetes mellitus: 1 = yes, 0 = no |
| Metabolic risk (w) | Binary | Waist status for assessing metabolic risk: 1 = yes, 0 = no |
| Metabolic risk (hyper) | Binary | Blood pressure for assessing metabolic risk: 1 = yes, 0 = no |
| Metabolic risk (fg) | Binary | Plasma fasting glucose level for assessing metabolic risk: 1 = yes, 0 = no |
| Metabolic risk | Binary | Plasma HDL-cholesterol for assessing metabolic risk: 1 = yes, 0 = no |
| Metabolic risk (triglycerides) | Binary | Plasma triglycerides for assessing metabolic risk: 1 = yes, 0 = no |
| Total Metabolic Syndrome Score | Integer | Total Metabolic Risk abnormalities score |
| Metabolic Syndrome at-risk level | Binary | Metabolic at-risk abnormalities level by score: 1 = yes, 0 = no |
| Hepatitis B Virus | Binary | Hepatitis B virus infection status: 1 = positive, 0 = negative |
| Hepatitis C Virus | Binary | Hepatitis C virus infection status: 1 = positive, 0 = negative |
| Chronic kidney disease (CKD) | Categorical | 0 = normal, 1 = mild, 2 = severe |
| Hepatitis B surface antigen level | Numeric | Hepatitis B surface antigen (HBsAg) in IU/mL |
| Hepatitis C virus antibody level | Numeric | Antibodies against hepatitis C virus (Anti-HCV) in S/CO ratio. |
| Hepatic steatosis | Binary | Historical documentation of steatosis by hepatic imaging (1 = yes, 0 = no) |
| *Lifestyle-Related Features:* |  |  |
| Diet | Categorical | 1 = omnivorous, 2 = vegan, 3 = vegetarian for breakfast only, 4 = others |
| Job type | Categorical | 0 = office-based, 1 = physically demanding. |
| Marital status | Categorical | 1 = single, 2 = married, 3 = divorced or separated, 4 = widowed. |
| Children | Ordinal | Number of children the patient has, categorized into ordered groups. Original numeric values of 4 or more were binned into an 'above 3' category to handle data sparsity. |
| Family support | Binary | Whether the person lives alone: 1 = yes, 0 = no |
| Self-care | Binary | Whether the person takes care of themselves: 1 = yes, 0 = no |
| Economic independence | Binary | Whether the person is financially independent: 1 = yes, 0 = no |
| Insurance status | Binary | Whether the person has additional insurance: 1 = yes, 0 = no. |
| Smoking status | Binary | Whether the person has ever smoked: 1 = yes, 0 = no. |
| Smoking cessation | Binary | Whether the person has ever quit smoking: 1 = yes, 0 = no. |
| Coffee consumption | Binary | Whether the person drinks coffee: 1 = yes, 0 = no. |
| Betel | Binary | Whether the person chew Betel nuts: 1 = yes, 0 = no. |
| RAND-36 Physical Functioning | Ordinal | RAND-36 subscale for physical functioning score, categorized from raw scores (0-100) into 5 levels using equal intervals of 20 points: 0=Low (0-19), 1=Mid-Low (20-39), 2=Medium (40-59), 3=Mid-High (60-79), 4=High (80-100). |
| RAND-36 Role Limitations | Ordinal | RAND-36 subscale for Role Limitations score, categorized from raw scores (0-100) into 5 levels using equal intervals of 20 points: 0=Low (0-19), 1=Mid-Low (20-39), 2=Medium (40-59), 3=Mid-High (60-79), 4=High (80-100). |
| RAND-36 Role Limitations-Emotional | Ordinal | RAND-36 subscale for Role Limitations (Emotional) score, categorized from raw scores (0-100) into 5 levels using equal intervals of 20 points: 0=Low (0-19), 1=Mid-Low (20-39), 2=Medium (40-59), 3=Mid-High (60-79), 4=High (80-100). |
| RAND-36 Energy/Fatigue | Ordinal | RAND-36 subscale for Energy/Fatigue score, categorized from raw scores (0-100) into 5 levels using equal intervals of 20 points: 0=Low (0-19), 1=Mid-Low (20-39), 2=Medium (40-59), 3=Mid-High (60-79), 4=High (80-100). |
| RAND-36 Emotional Well-Being | Ordinal | RAND-36 subscale for Emotional Well-Being score, categorized from raw scores (0-100) into 5 levels using equal intervals of 20 points: 0=Low (0-19), 1=Mid-Low (20-39), 2=Medium (40-59), 3=Mid-High (60-79), 4=High (80-100). |
| RAND-36 Social Functioning | Ordinal | RAND-36 subscale for Social Functioning score, categorized from raw scores (0-100) into 5 levels using equal intervals of 20 points: 0=Low (0-19), 1=Mid-Low (20-39), 2=Medium (40-59), 3=Mid-High (60-79), 4=High (80-100). |
| RAND-36 Pain | Ordinal | RAND-36 subscale for Pain score, categorized from raw scores (0-100) into 5 levels using equal intervals of 20 points: 0=Low (0-19), 1=Mid-Low (20-39), 2=Medium (40-59), 3=Mid-High (60-79), 4=High (80-100). |
| RAND-36 General Health | Ordinal | RAND-36 subscale for General Health score, categorized from raw scores (0-100) into 5 levels using equal intervals of 20 points: 0=Low (0-19), 1=Mid-Low (20-39), 2=Medium (40-59), 3=Mid-High (60-79), 4=High (80-100). |
| Anxiety level | Ordinal | HAD anxiety Score, raw numeric HADS score was categorized into 4 levels (0=Normal, 1=Mild, 2=Moderate, 3=Severe). |
| Depression level | Ordinal | HAD depression Score, raw numeric HADS score was categorized into 4 levels (0=Normal, 1=Mild, 2=Moderate, 3=Severe). |
| HAD | Ordinal | Total score from the Hospital Anxiety and Depression Scale (HADS), raw numeric total HADS score was categorized into 3 levels (0=Normal, 1=Moderate, 2=Severe). |
| Sleep quality | Ordinal | Sleep quality component of the Pittsburgh Sleep Quality Index (PSQI), levels: 0=None, 1=Mild, 2=Moderate, 3=Severe. |
| Sleep latency | Ordinal | Time taken to fall asleep, measured as a component of PSQI, levels: 0=None, 1=Mild, 2=Moderate, 3=Severe. |
| Sleep duration | Ordinal | During of total sleep time, measured as a component of PSQI, levels: 0=None, 1=Mild, 2=Moderate, 3=Severe. |
| Sleep efficiency | Ordinal | Sleep efficiency, measured as a component of PSQI, levels: 0=None, 1=Mild, 2=Moderate, 3=Severe. |
| Sleep disturbance | Ordinal | Frequency of sleep disturbances, measured as a component of PSQI, levels: 0=None, 1=Mild, 2=Moderate, 3=Severe. |
| Use of sleep medication | Ordinal | Use of sleep medication, measured as a component of PSQI, levels: 0=None, 1=Mild, 2=Moderate, 3=Severe. |
| Daytime dysfunction | Ordinal | Level of daytime dysfunction due to poor sleep, as a component of PSQI, levels: 0=None, 1=Mild, 2=Moderate, 3=Severe. |
| Mini Nutritional Assessment | Categorical | 24-30: normal, 17-23.5: at risk, <17: malnourished |
| AUDIT | Numeric | Alcohol Use Disorder Identification Test score |
| *Personal and Family History Features:* |  |  |
| Hypertension | Binary | Whether the person has hypertension: 1 = yes, 0 = no |
| Dysrhythmia | Binary | Whether the person has a history of arrhythmia: 1 = yes, 0 = no |
| Angina | Binary | Whether the person has a history of angina: 1 = yes, 0 = no |
| Myocardial infarction | Binary | Whether the person has a history of myocardial: 1 = yes, 0 = no |
| Hyperlipidemia | Binary | Whether the person has hyperlipidemia: 1 = yes, 0 = no |
| Heart failure (HF) | Binary | Whether the person has heart failure: 1 = yes, 0 = no |
| Endocrine disorders (endocrine) | Binary | Whether the person has endocrine disorders: 1 = yes, 0 = no |
| Thyroid disease | Binary | Whether the person has thyroid disease: 1 = yes, 0 = no |
| Gastritis | Binary | Whether the person has gastritis: 1 = yes, 0 = no |
| Hepatitis | Binary | Whether the person has hepatitis: 1 = yes, 0 = no |
| Fatty liver disease (FLD) | Binary | Whether the person has fatty liver disease: 1 = yes, 0 = no |
| Liver fibrosis (fibrosis) | Binary | Whether the person has liver fibrosis: 1 = yes, 0 = no |
| Cirrhosis | Binary | Whether the person has liver cirrhosis: 1 = yes, 0 = no |
| Polyposis | Binary | Whether the person has polyposis: 1 = yes, 0 = no |
| IBS | Binary | Whether the individual has irritable bowel syndrome (1 = yes, 0 = no) |
| parent – CVD | Binary | Whether the person’s parent has cardiovascular disease: 1 = yes, 0 = no |
| parent – Hype | Binary | Whether the person’s parent has hypertension: 1 = yes, 0 = no |
| parent - Dysrhythmia | Binary | Whether the person’s parent has a history of dysrhythmia: 1 = yes, 0 = no |
| parent - Angina | Binary | Whether the person’s parent has a history of angina: 1 = yes, 0 = no |
| parent - Myocardial infarction | Binary | Whether the person’s parent has a history of myocardial: 1 = yes, 0 = no |
| parent - Hyperlipidemia | Binary | Whether the person’s parent has hyperlipidemia: 1 = yes, 0 = no |
| parent - HF | Binary | Whether the person’s parent has heart failure: 1 = yes, 0 = no |
| parent - Diabetes | Binary | Whether the person’s parent has diabetes: 1 = yes, 0 = no |
| parent - Thyroid disease | Binary | Whether the person’s parent has thyroid disease: 1 = yes, 0 = no |
| Sibling - CVD | Binary | Whether the person’s sibling has cardiovascular disease: 1 = yes, 0 = no |
| Sibling - Hyper | Binary | Whether the person’s sibling has hypertension: 1 = yes, 0 = no |
| Sibling - Dysrhythmia | Binary | Whether the person’s sibling has dysrhythmia: 1 = yes, 0 = no |
| Sibling - Angina | Binary | Whether the person’s sibling has a history of angina: 1 = yes, 0 = no |
| Sibling - Myocardial infarction | Binary | Whether the person’s sibling has a history of myocardial: 1 = yes, 0 = no |
| Sibling - Hyperlipidemia | Binary | Whether the person’s sibling has hyperlipidemia: 1 = yes, 0 = no |
| Sibling - HF | Binary | Whether the person’s sibling has heart failure: 1 = yes, 0 = no |
| Sibling - Diabetes | Binary | Whether the person’s sibling has diabetes: 1 = yes, 0 = no |
| Sibling - Thyroid disease | Binary | Whether the person’s sibling has thyroid disease: 1 = yes, 0 = no |
| Child - CVD | Binary | Whether the person’s child has cardiovascular disease: 1 = yes, 0 = no |
| Child - Hyper | Binary | Whether the person’s child has hypertension: 1 = yes, 0 = no |
| Child - Dysrhythmia | Binary | Whether the person’s child has a history of dysrhythmia: 1 = yes, 0 = no |
| Child - Angina | Binary | Whether the person’s child has a history of angina: 1 = yes, 0 = no |
| Child - Myocardial infarction | Binary | Whether the person’s child has a history of myocardial: 1 = yes, 0 = no |
| Child - Hyperlipidemia | Binary | Whether the person’s child has a history of hyperlipidemia: 1 = yes, 0 = no |
| Child - Heart failure | Binary | Whether the person’s child has heart failure: 1 = yes, 0 = no |
| Child - Diabetes | Binary | Whether the person’s child has diabetes: 1 = yes, 0 = no |
| Child - Thyroid disease | Binary | Whether the person’s child has thyroid disease: 1 = yes, 0 = no |
| *Target Variable (to be predicted):* |  |  |
| An individual’s MAFLD status | Categorical | 0 = non-MAFLD, 1 = MAFLD non-diabetic, 2 = MAFLD diabetic |
