# Supplementary material for: Predicting Metabolic Dysfunction–Associated Fatty Liver Disease Phenotypes Among Adults: 2-Stage Contrastive Learning Method
Source: JMIR Med Inform. 2025 Dec 12;13:e75747. doi: 10.2196/75747 (PMC12702840; doi:10.2196/75747)
Supplement: Multimedia Appendix 2 [file medinform-v13-e75747-s002.docx]

**Appendix B: Key Hyperparameters of the Investigated Methods**

| Method | Hyper-Parameter Setting |
| --- | --- |
| Decision Tree | Maximum depth = 6; minimum samples per leaf = 5; minimum samples required to split an internal node = 10 |
| Random Forest | Number of estimators = 50; maximum depth = 8; minimum samples required to split an internal node = 10; minimum samples per leaf = 2, class weight = ‘balanced’ |
| XGBoost | Maximum depth = 3; minimum child weight = 5; number of estimators = 100 |
| MLP | Number of dense layers = 3; Layer dimensions = 16, 8, 8; Dropout rate = 0.1; Learning rate = 0.01; and Class weights = [0.2, 0.3, 0.4] |
| Autoencoder | Encoder dimensions = 32, 16; Encoding dimension = 8; Decoder dimensions = 16, 32; Classifier head = 8 → Num Classes; Dropout = 0.2; Learning rate = 0.001; and Class weights = [0.1, 0.3, 0.6] |
| GAT | Number of GAT layers = 2; Hidden dimension = 16; Attention heads = 4 (layer 1), 1 (layer 2); Dropout rate = 0.4; Learning rate = 0.001; and Class weights = [0.1, 0.3, 0.5] |
| GCN | Number of GCN layers = 2; GCN hidden dimensions = 32, 16; Classifier dimension = 8; Aggregation function = "add"; Dropout rate = 0.4; Learning rate = 0.01; and Class weights = [0.1, 0.3, 0.5] |
| GraphSAGE | Number of GraphSAGE layers = 2; Hidden dimension = 16; Dropout rate = 0.5; Learning rate = 0.01; and Class weights = [0.1, 0.3, 0.5] |
| Proposed Method | Unsupervised Stage Parameters: Number of GraphSAGE layers = 2; Dimension for node and edge embeddings = 32; Clinical fixed encoder architecture = 2 layers (Input→64→32); Lifestyle and genetics contrastive encoder architecture = 2 layers (Input→64→32); Contrastive Learning Batch size = 32; Temperature for contrastive loss = 0.1; and Learning rate = 0.001  Supervised Stage Parameters: Supervised classifier architecture = 2 dense layers (64→32); Dropout = 0.4; Two-stage fusion loss weight $\gamma$ =0.3, $\lambda$ =0.7; Learning rate = 0.005; and Class weights for loss function = [0.1, 0.5, 0.6]. |

Notes: All deep learning-based methods use Adam optimizer; all neural network-based methods use ReLU activation functions, with batch normalization applied and Final Activation = Softmax.
